# Methionine addition improves the acid tolerance of *Lactiplantibacillus plantarum* by altering cellular metabolic flux, energy distribution, lipids composition

**DOI:** 10.1007/s44154-022-00072-z

**Published:** 2022-11-14

**Authors:** Qiang Meng, Yueyao Li, Yuxin Yuan, Shaowen Wu, Kan Shi, Shuwen Liu

**Affiliations:** 1 College of Enology, Northwest A&F University, Yangling, 712100 Shaanxi China; 2 Shaanxi Engineering Research Center for Viti-Viniculture, Yangling, 712100 Shaanxi China; 3 Viti-viniculture Engineering Technology Center of State Forestry and Grassland Administration, Yangling, 712100 Shaanxi China; 4 Heyang Experimental and Demonstrational Stations for Grape, Northwest A&F University, Heyang, 715300 Shaanxi China; 5 Ningxia Helan Mountain’s East Foothill Wine Experiment and Demonstration Station, Northwest A&F University, Yongning, 750104 Ningxia China

**Keywords:** Acid stress, *Lactiplantibacillus plantarum*, Methionine, Cell viability, Transcription level, Membrane lipids

## Abstract

This paper reported a wine-derived lactic acid bacterium, *Lactiplantibacillus plantarum* XJ25, which exhibited higher cell viability under acid stress upon methionine supplementation. Cellular morphology and the composition of the cytomembrane phospholipids revealed a more solid membrane architecture presented in the acid-stressed cells treated with methionine supplementation. Transcriptional analysis showed *L. plantarum* XJ25 reduced methionine transport and homocysteine biosynthesis under acid stress. Subsequent overexpression assays proved that methionine supplementation could alleviate the cell toxicity from homocysteine accumulation under acid stress. Finally, *L. plantarum* XJ25 employed energy allocation strategy to response environmental changes by balancing the uptake methionine and adjusting saturated fatty acids (SFAs) in membrane. These data support a novel mechanism of acid resistance involving methionine utilization and cellular energy distribution in LAB and provide crucial theoretical clues for the mechanisms of acid resistance in other bacteria.

**Supplementary Information:**

The online version contains supplementary material available at 10.1007/s44154-022-00072-z.

## Introduction

Malolactic fermentation (MLF) conducted by lactic acid bacteria (LAB) is an essential strategy for biological deacidification in food processing (Markkinen et al. 2019; Tiitinen et al. 2007; Virdis et al. 2021). Malic acid in fruit juice, vegetable juice or wine can be converted to lactic acid after MLF, resulting in an improvement in the flavor and mouthfeel of the final product (Fonseca et al. 2022; Lombardi et al. 2020; Tomita et al. 2017). On the other hand, shelf-life of the product was extended attributing to increased antimicrobial compounds after MLF (Muhialdin et al. 2020, 2021). LAB from genus *Lactiplantibacillus*, especially *Lactiplantibacillus plantarum*, have been widely utilized as the starter of MLF in recent researches owing to its good performance in the consumption of L-malic acid and release of variety aroma compound (Brizuela et al. 2018, 2021; Hernandez et al. 2007; Lucio et al. 2017; Markkinen et al. 2022; Sun et al. 2016). However, many adverse factors inhibited *Lactiplantibacillus* growth during fermentation especially acid stress (Sun et al. 2016).

Weak acids hardly dissociate and can directly penetrate through the lipid bilayer of the cytomembrane, they usually disrupt the balance of intracellular pH and decrease the activity of biochemical enzymes (Lund et al. 2014; Yang et al. 2019). Several publications have reported the mechanisms of acid resistance in food microorganisms. It has been revealed that glutamine deamidation played a vital role in the acid resistance of *L. reuteri* in 100 mmol/L lactate buffer (pH 3.5) (Teixeira et al. 2014). This deamidation action also contributed to the acid resistance of *L. reuteri* in 50 mmol/L sodium acetate buffer (pH 4.5) (Li et al. 2020). The transcriptional upregulation of lactate catabolism, H^+^ extrusion, and glycerol transport genes was crucial in the co-culture of *Pichia kudriavzevii* and *Saccharomyces cerevisiae* in response to high lactic acid stress (40 g/L) during Baijiu fermentation (Deng et al. 2020). Other researches reported an increased level of Sam2p (an enzyme catalyzing the conversion of methionine to *S*-adenosylmethionine) and the lethal effect of the overexpression of *SAM2* in *S. cerevisiae* BY4741 after lactic acid exposure (Dato et al. 2014). Interestingly, exogenously supplied D-methionine improved the hydrochloric acid tolerance of *Lactococcus lactis* by changing the composition of the cell wall (Wu et al. 2020). These suggested elaborate adjustments of amino acid metabolism in microbes under acid stresses.

The affinity-based transport of methionine determined the phenotypic heterogeneity in *L. lactis* at the single-cell level when the extracellular methionine supply was limited (Hernandez-Valdes et al. 2020). The number and genetic organization of methionine transporters vary in different bacterial genomes (Hullo et al. 2004; Zhang et al. 2003). These clues indicated that the methionine uptake of a certain strain under a given nutrient condition was complicated by specific adaptation and regulation. Considerable data were analyzed to characterize the potential methionine metabolic pathways and their regulatory systems using computational methods (Liu et al. 2012; Rodionov et al. 2004). Such efforts paved the way for the exploration of genetic diversity in methionine utilization (Wuthrich et al. 2018a) and altered metabolic fluxes upon perturbation (Veith et al. 2015) as well as the selection of desirable LAB strains for the food industry (Wu and Shah 2018; Wuthrich et al. 2018b). Our latest research screened a random amplified polymorphic DNA marker in wine-derived *L. plantarum* strains, S116–680 (a part of the nucleotide sequence of a putative methionine transport protein, MTP), which was shared by the acid-resistant strains but was absent in the acid-sensitive strains, speculating that the presence of this marker gene was associated with the hydrochloric acid resistance of *L. plantarum* (Liu et al. 2015). Fortunately, we found this gene in the genome of *L. plantarum* XJ25 (NCBI accession number: NZ_CP068448.1), a LAB strain isolated from Chinese red wine (Zhao et al. 2016).

To further explore the biological function of this methionine transport protein in *L. plantarum* under acid stress, we started with the construction of a mutant XJ25Δ*mtp* and monitored the cell growth of the wild-type XJ25 and the mutant XJ25Δ*mtp* in this study. The tolerance of wine-derived *L. plantarum* cells to acid stress in a chemical determined medium (CDM) was examined. The effects of L-methionine to *L. plantarum* cells under acid tress were also studied, including changes in cell viability and cellular surface morphology, GTP levels, membrane phospholipids as well as the expression of genes related to methionine uptake and metabolism.

## Results and discussion

### Methionine supplement increases the acid tolerance of *L. plantarum* in a CDM

**Fig. 1:**
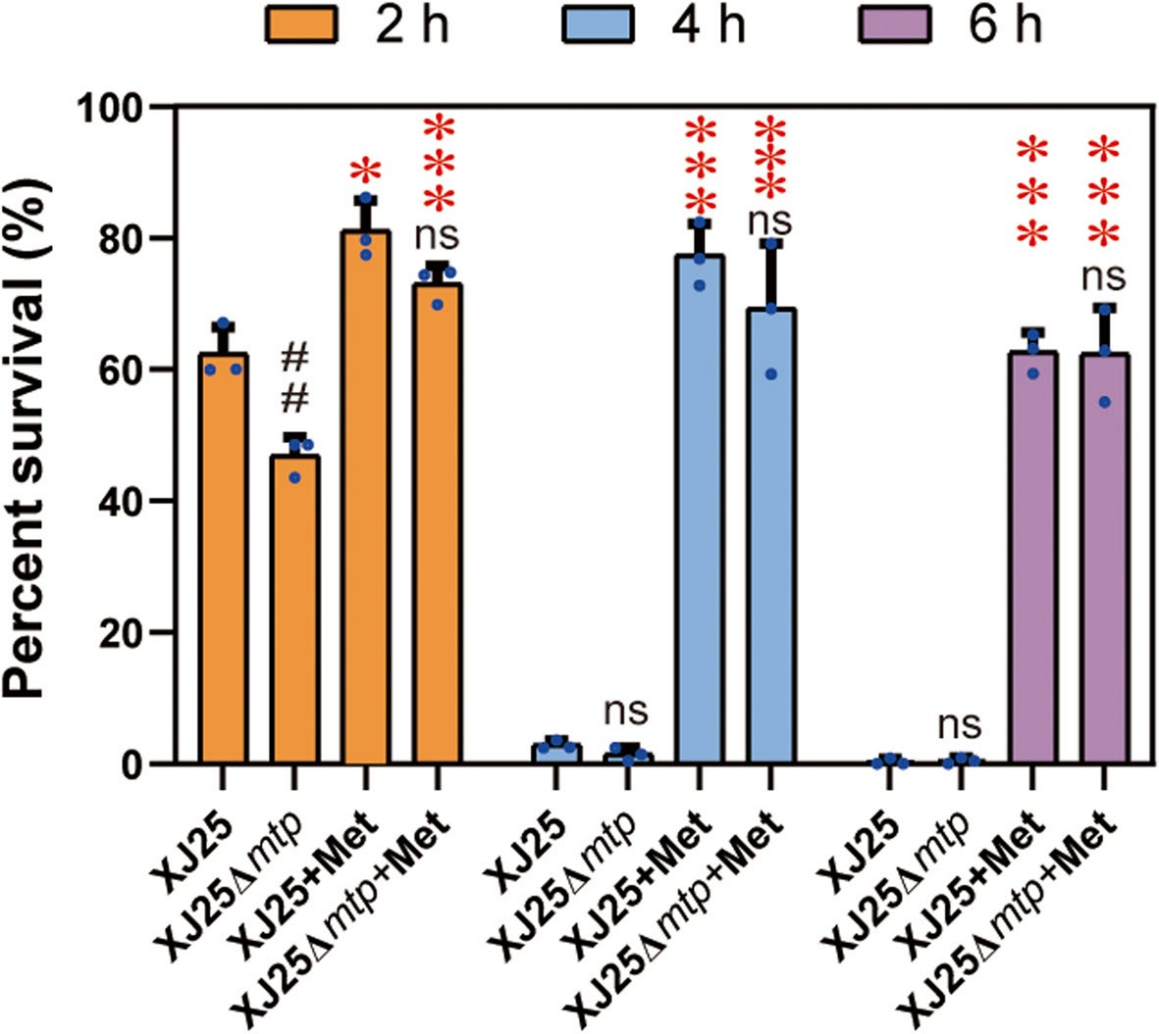
Effects of acid stress on the survival rates of the wild-type XJ25 and the mutant XJ25Δ*mtp* cells in CDM. + Met, 2 mM methionine. ^## ^*p* < 0.01, ns, not significant (*p* > 0.05), comparison between the wild-type XJ25 and the mutant XJ25Δ*mtp* (XJ25 vs XJ25Δ*mtp*, XJ25 + Met vs XJ25Δ*mtp* + Met, Student’s *t*-test); * *p* < 0.05, *** *p* < 0.001, comparison between the stressed cells supplied with and without methionine (XJ25 vs XJ25 + Met, XJ25Δ*mtp* vs XJ25Δ*mtp* + Met, Student’s *t*-test)

The pH of medium was adjusted using malic acid owing to the fact that it is the top two abundant acids in wines desired for MLF, and that on the other hand, malic acid stress was significant among other acids treatments (Fig. 1 and Fig. S1). To illustrate the potential influence of amino acids on the cell viability, *mtp* was knockouted from the genome of *L. plantarum* XJ25 and an amino acid-deficient medium (namely CDM) was applied to assay the physiological role of *mtp* under acid stress. In CDM, the disruption mutant exhibited lower cell viability than the wild-type strain under acid stress without methionine supplementation at 2 h but similar survival rates at 4 h or 6 h (Fig. 1). When the acidic CDM (pH 3.2) was supplied with 2 mmol/L methionine, the XJ25 cells and XJ25Δ*mtp* cells showed significantly higher acid tolerance than the untreated counterpart in the two acid stress assays (Fig. 1). Moreover, this increased cell viability after the methionine supplementation was also observed in other organic acid stress assays, such as tartaric, lactic, citric, and lactic acid stress (Fig. S1). These results suggested that the methionine supplementation was essential for the better survival of *L. plantarum* under acid stress in CDM. Interestingly, the XJ25- and XJ25Δ*mtp*-stressed cells supplied with methionine showed no significant difference in cell viability under acid stress (Fig. 1), indicating that MTP might not be involved in the protective effect of methionine and unknown acid responses of the XJ25Δ*mtp*-stressed cells had been activated and compensated for the effects of the deletion of *mtp* under this stress. However, this compensation effect was not observed in other acid stress assays (Fig. 1 and Fig. S1), implying a special response mechanism of *L. plantarum* XJ25 under acid stress.

### Methionine supplement protects cell membrane of *L. plantarum* XJ25

**Fig. 2:**
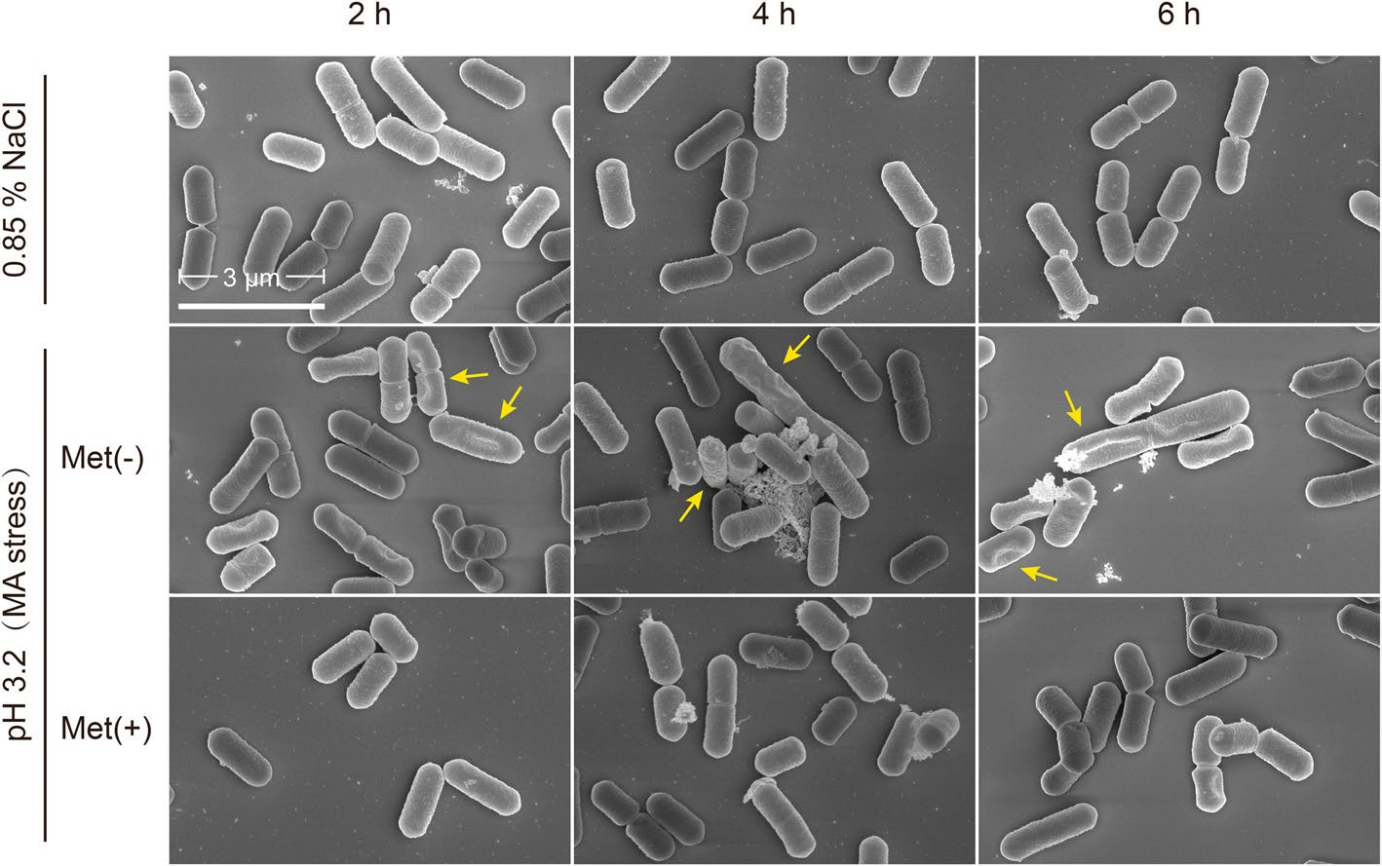
Scanning electron microscopy of *L. plantarum* XJ25 cells under acid stress. Met(−), without methionine; Met(+), with 2 mM methionine. Scale bar represents 3 μm

To directly explore the physiological changes of the XJ25 cells induced by acid stress, we next examined the subtle differences in cellular morphology and the composition of the phospholipids between cells treated with and without methionine supplementation by scanning electron microscopy and GC-MS, respectively. As shown in Fig. 2, cells without methionine supplementation under acid stress exhibited apparent cell shrinking, sags, crests and even collapsing, while methionine supplementation provided the stressed cells with smoother, plump and intact cellular surface morphology.

The fatty acid composition of cell membrane was assayed to explain the effect of methionine supplementation on XJ25 cells. The major membrane lipids of the stressed cells were myristic acid (C14:0), palmitic acid (C16:0), stearic acid (C18:0), cyclopropanes dihydrosterculic acid and lactobacillic acid (C19:0cyc), palmitoleic acid (C16:1), oleic acid (C18:1) and linoleic acid (C18:2) (Table 1). These seven fatty acids represented more than 95% of the total fatty acids of the XJ25-stressed cells. Cells under acid stress for 2 h did not largely change the fatty acid profiles compared with those of control cells without acid stress. However, the saturated fatty acids/unsaturated fatty acids ratio (SFAs/UFAs ratio) increased from 1.37 in XJ25 cells stressed for 2 h to 1.69 in cells stressed for 4 h. Specifically, the SFAs C16:0 and C18:0 of the stressed cells at 4 h showed a significant increase, while the UFA C18:1 exhibited a significant decrease compared with that at 2 h. In a previous study, increased SFAs and decreased UFAs were observed in acid-adapted *L. casei* cells, which displayed higher cell viability than cells treated without acid adaptation (Broadbent et al. 2010). A similar transition of membrane lipids was also observed in *L. plantarum* ZDY2013 under acid stress (Huang et al. 2016). Interestingly, the SFAs/UFAs ratio of stressed cells supplemented with methionine at 2 h was also significantly higher than that of cells without methionine supplementation, while this difference disappeared at 4 h (Table 1). These observations collectively demonstrated that the delicate transition of membrane lipids from UFAs to SFAs was a pivotal strategy adopted by lactobacilli cells in answering acid stress and nutrient supplementation.

**Table 1:** Effect of the methionine supply on the fatty acid composition of the membrane phospholipids of *L. plantarum* XJ25 under acid stress

Fatty acids or parameters	0 h	2 h	2 h + Met	4 h	4 h + Met
C14:0	2.33 ± 0.02^a^	2.19 ± 0.21^ab^	2.00 ± 0.03^b^	2.32 ± 0^a^	2.30 ± 0.04^a^
C16:0	30.63 ± 0.49^b^	29.69 ± 1.27^b^	29.56 ± 0.42^b^	33.29 ± 0.32^a^	34.13 ± 0.18^a^
C18:0	12.78 ± 0.09^c^	12.37 ± 0.21^c^	16.40 ± 0.23^a^	15.97 ± 0.48^a^	15.17 ± 0.12^b^
C19:0cyc	10.35 ± 0.15^a^	10.85 ± 0.15^a^	9.99 ± 0.13^a^	9.32 ± 0.42^a^	10.36 ± 1.21^a^
C16:1	2.99 ± 0.08^a^	2.83 ± 0.31^ab^	2.73 ± 0.04^ab^	2.52 ± 0.01^b^	2.60 ± 0.08^ab^
C18:1	35.41 ± 0.33^a^	36.09 ± 0.54^a^	34.95 ± 0.43^a^	31.94 ± 0.69^b^	33.22 ± 0.74^b^
C18:2	1.31 ± 0.02^a^	1.39 ± 0.08^a^	1.42 ± 0.09^a^	1.71 ± 0.46^a^	1.52 ± 0.06^a^
SFAs^**I**^	56.07 ± 0.41^bc^	55.09 ± 1.53^c^	57.94 ± 0.55^b^	60.89 ± 0.38^a^	61.95 ± 0.87^a^
UFAs^**II**^	39.7 ± 0.44^a^	40.31 ± 0.76^a^	39.09 ± 0.48^a^	36.16 ± 0.22^b^	37.34 ± 0.88^b^
SFAs/UFAs ratio	1.42 ± 0.01^c^	1.37 ± 0.01^c^	1.49 ± 0.04^b^	1.69 ± 0.02^a^	1.66 ± 0.01^a^

Values with different letters indicate statistically significant differences (*p* < 0.05, one-way ANOVA with Tukery’s test)

^I^*SFAs* Saturated fatty acids include C14:0, C16:0, C18:0 and C19:0cyc

^II^*UFAs* Unsaturated fatty acids include C16:1, C18:1 and C18:2

### Methionine supplement neutralizes the cell toxicity under acid stress

**Fig. 3:**
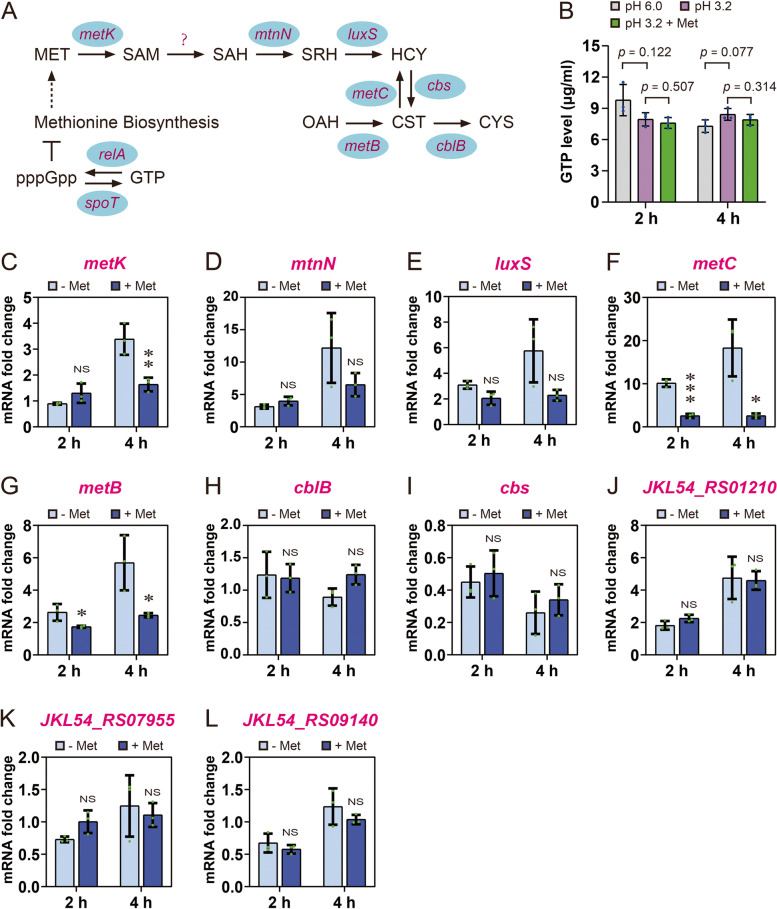
Methionine biosynthesis and metabolic pathway in *L. plantarum* XJ25 (**A**). MET, methionine; SAM, *S*-adenosylmethionine; SAH, *S*-adenosylhomocysteine; SRH, *S*-ribosylhomocysteine; HCY, homocysteine; CST, cystathionine; CYS, cysteine; OAH, *O*-acetylhomoserine. The question mark indicated that the gene required for the biochemical reaction was not found in the genome of *L. plantarum* XJ25. Solid and dashed line arrow indicates a direct and indirect pathway, respectively. T-type arrow indicates an inhibitory effect. GTP levels of *L. plantarum* XJ25 under acid stress with and without the methionine supplementation (**B**). + Met, 2 mM methionine. The numbers above the columns represent the statistical significances between two group comparisons (Student’s *t*-test).The mRNA levels of *metK* (**C**), *mtnN* (**D**), *luxS* (**E**), *metC* (**F**), *metB* (**G**), *cblB* (**H**), *cbs* (**I**), *JKL54_RS01210* (**J**), *JKL54_RS07955* (**K**), *JKL54_RS09140* (**L**) in the acid-stressed cells of *L. plantarum* XJ25*.* + Met, 2 mM methionine. * *p* < 0.05, ** *p* < 0.01, *** *p* < 0.001, NS, not significant (*p* > 0.05), comparison between the treatments with and without methionine supplementation

**Fig. 4:**
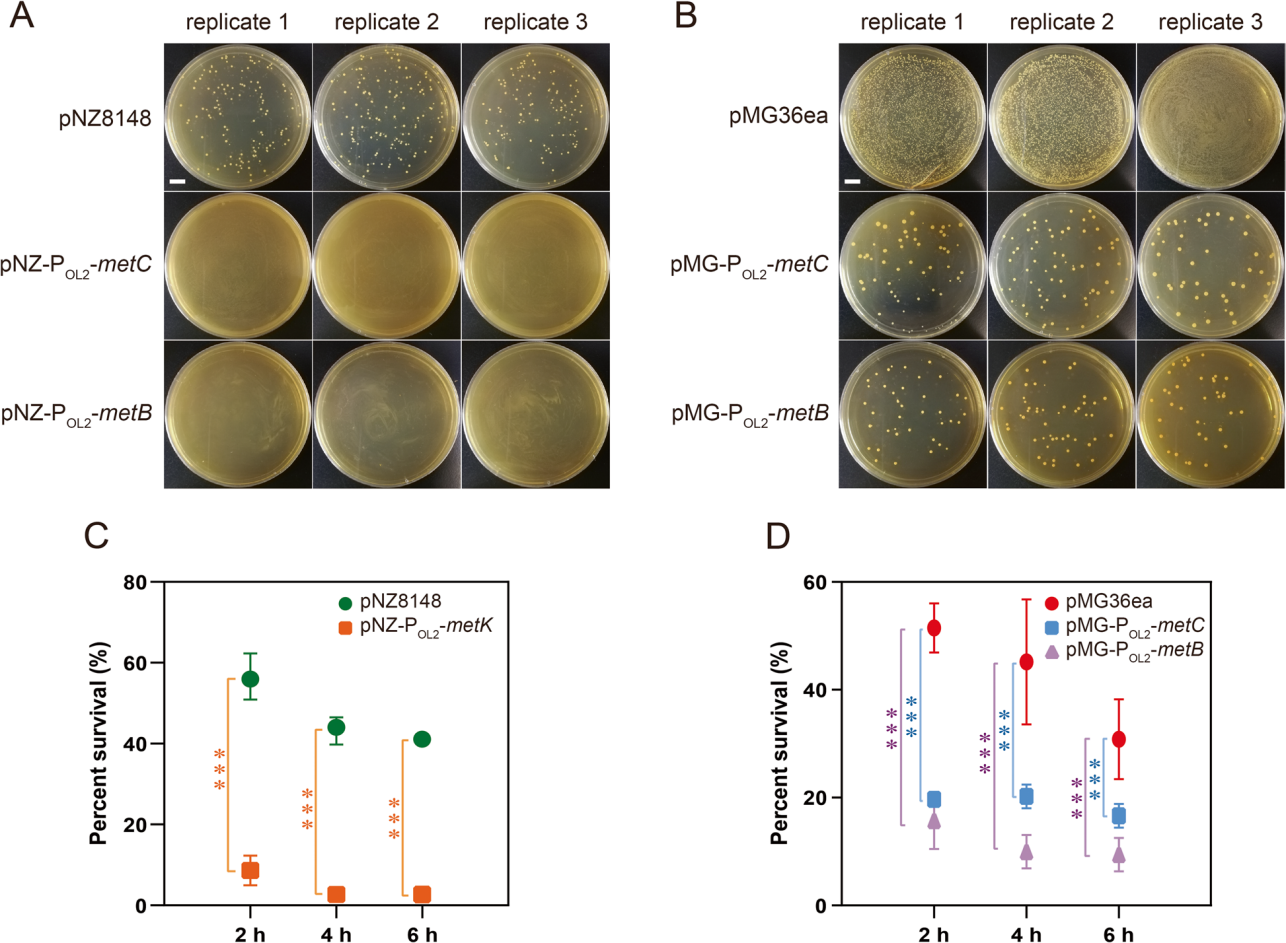
Plate screening after the *L. plantarum* XJ25 competent cells electrotransformed with the pNZ8148 plasmid derivatives, which shared a high-copy-number replicon in their backbones (**A**). Scale bar represents 1 cm. Plate screening after the *L. plantarum* XJ25 competent cells electrotransformed with the pMG36ea plasmid derivatives, which harbored a medium-copy-number replicon in their backbones (**B**). Scale bar represents 1 cm. The survival rates of *L. plantarum* XJ25 cells overexpressing *metK* (**C**), *metC* and *metB* (**D**) under acid stress with 2 mM methionine. *** *p* < 0.001 (Student’s *t*-test)

To further explain the protection of methionine to *L. plantarum* XJ25 under acid stress, the changes in expression levels of genes in the methionine metabolic pathway of *L. plantarum* XJ25 were analyzed. Methionine can be adenylated to produce *S*-adenosylmethionine, a universal methyl group donor to metabolites, proteins and nucleic acids during cell metabolism, conducted by *S*-adenosylmethionine synthetase encoded by *metK* (Fig. 3A) (Lieber and Packer 2002). After exposure to acid for 2 h, the expression level of *metK* in XJ25 cells showed nearly no change between the methionine treatment and the control group (without methionine) (Fig. 3C). At 4 h, *metK* in XJ25 cells of the methionine treatment was downregulated 2.07-fold compared with that of the control group. Similar downregulation effects were also observed in the transcription levels of *metC* and *metB* (Fig. 3F and G), which encode cystathionine gamma synthase and cystathionine beta lyase, respectively. These three genes are involved in homocysteine biosynthesis (Fig. 3A). The transcription of *metN*, *luxS*, *cblB* and *cbs* in XJ25 cells under acid stress with methionine remained unchanged compared with that of the untreated group (Fig. 3D, E, H and I). However, the transcription level of *metK*, *metN*, *luxS*, *metC*, *metB* under acid stress was upregulated 3.38-, 12.18-, 5.76-, 18.29-, 5.29-fold, respectively, after 4 h compared to the nonacid treatment (Fig. 3C, D, E, F and G). These results suggested that acid stress induced the accumulation of homocysteine in XJ25 cells, possibly resulting in a toxic effect on cell viability (Fig. 1). Interestingly, methionine supplementation during stress could alleviate this toxic effect by decreasing the flux of homocysteine biosynthesis to a certain extent (Fig. 3C, F and G), thus leading to higher viability (Fig. 1). To prove this toxic effect, we constructed three overexpression strains, namely XJ25/pNZ-P_OL2_-*metK*, XJ25/pNZ-P_OL2_-*metC *and XJ25/pNZ-P_OL2_-*metB*, to examine the effects of a constitutively enhanced homocysteine biosynthesis on the cell viability under acid stress in the presence of exogenous methionine. During the recombinant construction, no transformant could be obtained by electroporation with the pNZ-P_OL2_-*metC* plasmid or the pNZ-P_OL2_-*metB* plasmid (Fig. 4A), which shared a high-copy-number replicon, and the P_OL2_-*metC* and P_OL2_-*metB* expression cassette had to be inserted into a medium-copy-number plasmid (pMG36ea) for recombinant screening (Fig. 4B). As expected, the *metK*, *metC* and *metB* overexpression strain showed significantly decreased cell viability under acid stress with methionine supplementation (Fig. 4C and D). These results together indicated severe cell toxicity resulting from the increased flux of homocysteine biosynthesis under acid stress in the absence of exogenous methionine and a rescuing effect contributed by exogenous methionine.

### Acid stress triggers severe stringent response

As shown in Fig. 3A, guanosine pentaphosphate (pppGpp) during the stringent response could enable rational energy allocation for bacterial survival and adaptation by repressing amino acid biosynthesis (Irving et al. 2021; Kriel et al. 2014; Qi et al. 2021). This response decreases intracellular GTP level, while GTP played vital roles in multi-stress resistance (Ryssel et al. 2014). In this study, no significant difference was observed in the GTP content of the XJ25 cells under acid stress and control condition (Fig. 3B), meanwhile methionine supplementation did not impact the GTP levels (Fig. 3B). The *JKL54_RS01210* locus, the *JKL54_RS07955* locus and the *JKL54_RS09140* locus are three *relA*/*spot* analogs. RelA/SpoT can catalyze the conversion of GTP to pppGpp when cells suffer from nutrient deprivation (Irving et al. 2021). The transcription levels of these three loci (Fig. 3J, K and L) were consistent with those observed in the intracellular GTP levels. We inferred that a severe stringent response of the cells was activated because the cells exhibited an extremely low concentrations of GTP (Fig. 3B, gray columns), even acid stress cannot exacerbate the stringent response of the XJ25 (Fig. 3B, purple columns). Therefore, the accumulated alarmone (pppGpp) caused the stressed cells to suppress methionine biosynthesis (Fig. 3A). High pppGpp pool leading to an increase in survival was also observed in acid-stressed *Oenococcus oeni* (Qi et al. 2021) and *Alicyclobacillus acidoterrestris* (Zhao et al. 2022).

### *L. plantarum* XJ25 provokes energy allocation strategy to acid stress

**Fig. 5:**
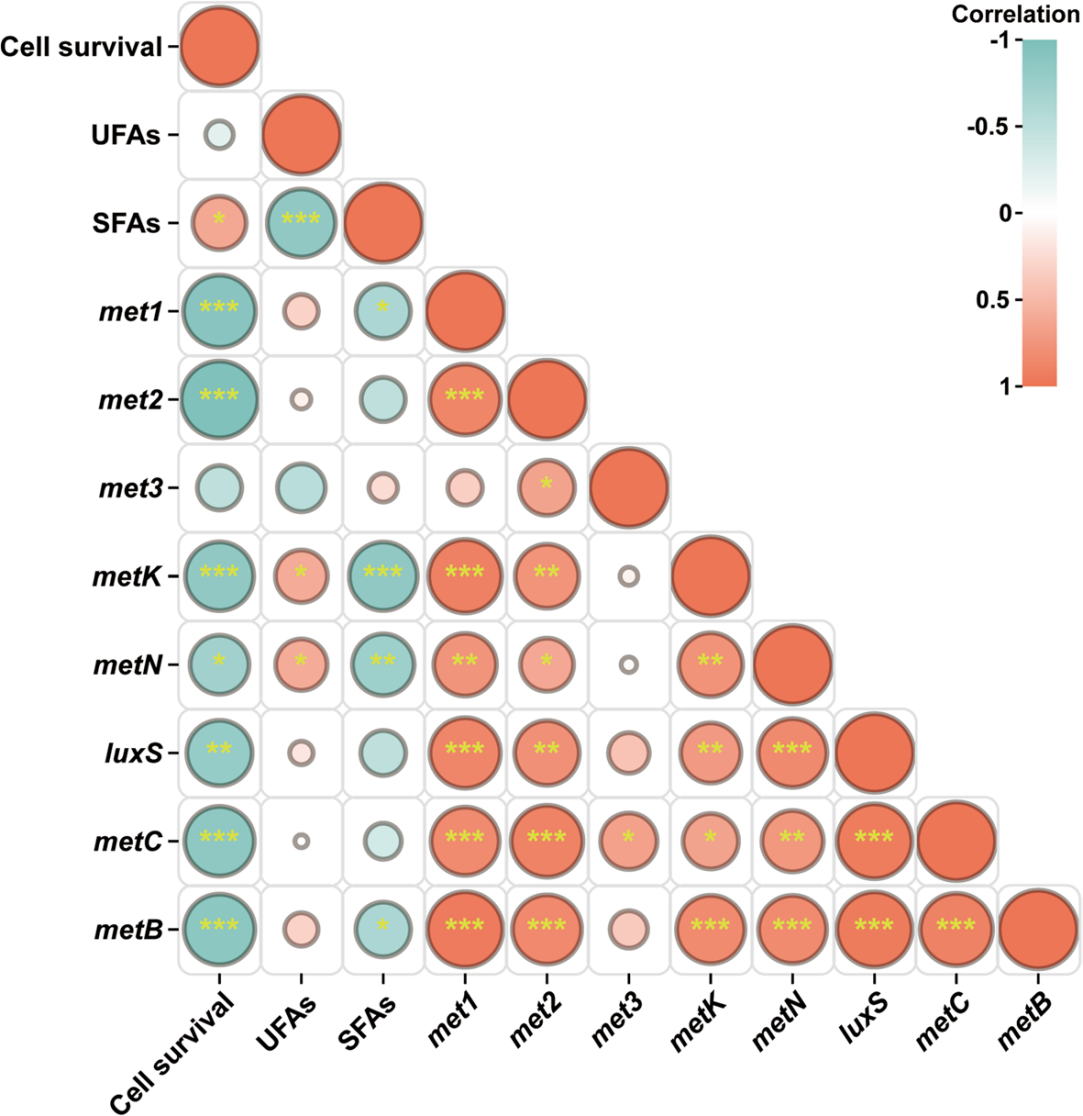
Correlation analysis of cell viability, membrane lipids and the transcription levels of genes in the methionine metabolic pathway in this study. The mRNA levels of *met1*, *met2*, *met3* were visualized in Fig. S2. * *p* < 0.05, ** *p* < 0.01, *** *p* < 0.001 (Student’s *t*-test)

**Fig. 6:**
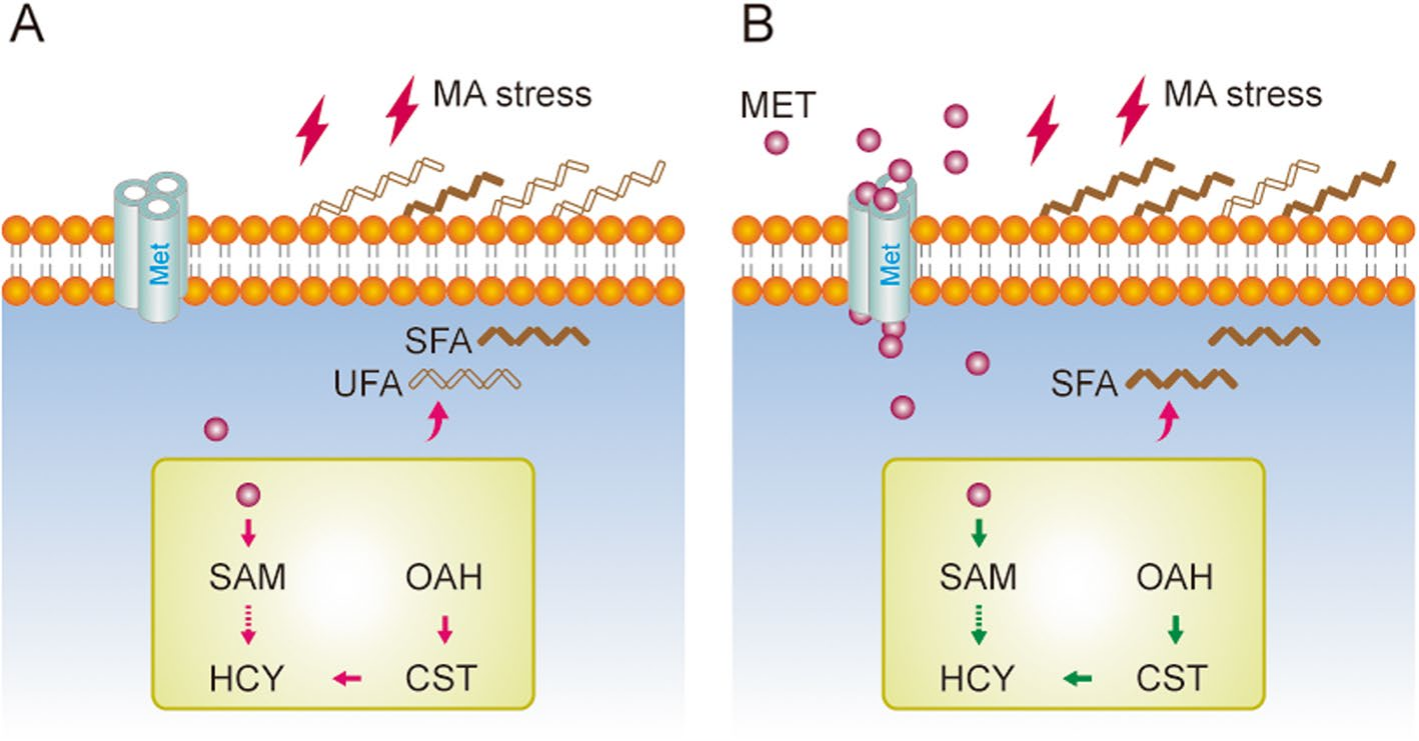
A regulatory mechanism of *L. plantarum* responding to acid stress without (**A**) and with methionine supplementation (**B**). Met indicates the methionine transporter in *L. plantarum* XJ25. MA, malic acid; SFA, saturated fatty acid; UFA, unsaturated fatty acid; MET, methionine; SAM, *S*-adenosylmethionine; HCY, homocysteine; CST, cystathionine; OAH, *O*-acetylhomoserine. Pink and green arrows indicate enhanced and decreased fluxes, respectively. Solid line arrows represent direct effects, dashed and flexual line arrows represent indirect effects

Next, we performed Pearson correlation analysis to further decipher the relationship among cell viability, membrane lipids and the transcription levels of genes in the methionine metabolic pathway in this study. As shown in Fig. 5, the cell viability was significantly positively correlated with the contents of SFA, but negatively correlated with the transcription levels of genes in methionine utilization. Moreover, the transcription levels of genes in methionine utilization showed a high positive correlation among each other. These observations might imply an energy allocation strategy employed by *L. plantarum* XJ25 during acid stress or an environmental shift. For example, when extracellular methionine was absent or scarce, the surviving cells had to allocate more cellular energy to upregulate the gene expression of methionine uptake and metabolism (Fig. 6A). The resulting effect of unbalanced energy allocation caused metabolic disorders and weakened the levels of membrane SFAs required for cell survival (Fig. 6A). When the methionine supply was abundant, the stressed cells possessed a more reasonable cellular energy redistribution in methionine utilization and a more solid membrane structure (Fig. 6B), thus displaying better resistance and cell vitality against acid stress.

## Conclusion

In summary, acid stress (pH 3.2) significantly damaged cell integrity, induced the accumulation of homocysteine which could produce cell toxicity, and decreased *L. plantarum* viability, while MTP might not play vital role in acid stress response. Methionine supplementation could improve acid resistance of *L. plantarum* cells by adjusting SFAs/UFAs ratio of cell membrane to maintain cell structure, changing methionine metabolic flux to alleviate toxic effect produced by homocysteine accumulation. In addition, the analysis of GTP levels indicated acid stress induced severe stringent response, and that cells applied energy allocation strategy to resist to stress or nutrient deficiencies. These findings revealed a novel mechanism of acid resistance involving methionine utilization and cellular energy distribution, which provided crucial clues for the molecular mechanisms of acid resistance in other bacteria.

## Materials and methods

### Strains and culture conditions

All strains used in this study are listed in Table S1. *Escherichia coli* strains were grown at 37 °C in Luria-Bertani (LB) broth (Solarbio, Beijing, China). *L. plantarum* strains were cultured in de Man-Rogosa-Sharpe (MRS) broth (Solarbio, Beijing, China) at 37 °C. Kanamycin (50 mg/L), erythromycin (40 mg/L), or chloramphenicol (10 mg/L) were supplemented into the media necessarily. CDM was used for the acid stress assays, constituents were as follows (per liter): yeast nitrogen base (without amino acids; Solarbio, Beijing, China), 6.7 g; (NH_4_)_2_SO_4_, 5 g; glucose, 10 g; adenine, uracil, thymine, guanine, and cytosine, 10 mg each; the final pH was adjusted to 3.2 or 6.0 by 8.4 × 10^− 3^ mol/L malic acids and 3 mol/L NaOH, respectively.

### DNA manipulation and mutant construction

The cell lysate obtained using Lysis Buffer for Microorganisms (Takara, Dalian, China) to Direct PCR according to the instructions was used as a template for PCR. The knockout plasmid pLCNICK-Δ*mtp* was constructed as described by Song et al. (2017). Detailed procedures were as follows: two homologous arms flanking the open reading frame of *mtp* were amplified by the primers mtp-uF/mtp-uR and mtp-dF/mtp-dR, respectively (Table S2); a short DNA sequence, which transcribed a single guide RNA (sgRNA) targeting *mtp* in the genome of *L. plantarum* XJ25, was amplified by the primers sg-F/sg-mtp-R. These three fragments were connected together by Gibson assembly, generating a linear DNA named Δ*mtp*-sgRNA. Then, the linear Δ*mtp*-sgRNA was cloned into the plasmid pLCNICK between the *Apa* I and *Xba* I sites using One Step PCR Cloning Kits (Novoprotein, Shanghai, China) according to the manufacturer’s instructions to produce the knockout plasmid pLCNICK-Δ*mtp*. Overexpression plasmids were constructed as follows: the open reading frames of *metK*, *metC*, and *metB* were amplified by the primers pNZ-metK-F/pNZ-metK-R, pNZ-metC-F/pNZ-metC-R, andpNZ-metB-F/pNZ-metB-R, respectively; these linear products were ligated with the linear vector pNZ-P_OL2_ (amplified by the primers pNZ-zt-F/pNZ-zt-R) to produce the overexpression plasmids pNZ-P_OL2_-*metK*, pNZ-P_OL2_-*metC*, and pNZ-P_OL2_-*metB*, respectively. The constructs pMG-P_OL2_-*metC* and pMG-P_OL2_-*metB* were also obtained through this method (using the pMG36ea plasmid as the expression skeleton).

The recombinant constructs described above were transformed into the *Escherichia coli* HST04 *dam*^*−*^/*dcm*^*−*^ strain (Takara, Dalian, China) for enrichment. Then, these recombinant plasmids were extracted using the Plasmid Mini Kit I (Omega, Guangzhou, China) and introduced into *L. plantarum* XJ25 competent cells according to the optimized electroporation protocol previously reported (Meng et al. 2021). The *L. plantarum* XJ25 recombinant harboring the plasmids pLCNICK-Δ*mtp*, was streaked on MRS plates without erythromycin for plasmid curing after the expected mutation was confirmed, yielding the mutant XJ25Δ*mtp.*

### Cell survival under acid stress

The acid stress experiments were performed as follows: ten microliters of overnight cultures were inoculated into 10 mL of fresh MRS broth and then incubated at 37 °C. Cells were immediately centrifuged at 1000 *g* for 5 min when the optical density of 600 nm (OD_600_) reached ~ 0.3. Then, the cells were washed three times with sterile saline (0.85% NaCl, w/v) and finally suspended in CDM (pH 3.2 or pH 6.0) with or without 2 mmol/L methionine (L-methionine, if not stated). The suspensions were serially diluted with sterile saline (0.85% NaCl, w/v), spread on MRS plates after incubated at 37 °C for 2 h, 4 h or 6 h, then incubated for 2 days for enumeration. Cell survival was calculated by the following formula:

$$\text{Cell}\;\text{survival}=\frac{{\text{CAS}}_{\mathrm i}}{{\text{CNAS}}_0}\times100\%$$

CAS_*i*_ represents the number of colonies obtained after *i* h in the acid stress treatment (pH 3.2) with or without 2 mmol/L methionine, while CNAS_*0*_ represents the number of colonies obtained at 0 h in the nonacid stress treatment (pH 6.0).

### Scanning electron microscopy

Samples for scanning electron microscopy were prepared according to Wang et al. (2019) method without any modifications.

### Extraction and determination of cytomembrane fatty acids

The total membrane lipids were extracted, methylesterified and detected following previously reported methods with some modifications (Ingham et al. 1998; Xu et al. 2020). Briefly, cells were collected by centrifugation at 4 °C (8000 *g*, 10 min). The pellets were suspended in 1 mL of 0.5 M sodium methoxide and heated at 100 °C for 10 min, then dosed with 1 mL of BF_3_-methanol (15% BF_3_, w/v) after cooling to room temperature, mixed thoroughly by vortexing, the mixture was heated at 100 °C for 10 min and cooled to room temperature. The resulting solution was extracted with 1 mL n-hexane and detected with a TRACE™ 1310 GC–MS (Thermo Fisher Scientific, Waltham, MA, USA; Column: WM-WaxMS, Shanghai, China). The relative abundance of each membrane lipid was calculated according to the method described by Xu et al. (2020).

### Transcriptional analysis of genes in the methionine metabolic pathway

Cells were collected by centrifugation at 4 °C (8000 *g*, 10 min), the supernatant was discarded, and the pellet was immediately stored at − 80 °C. The *AG RNAex Pro* Reagent and SYBR Green Premix *Pro Taq* HS qPCR Kits (Accurate Biology, Changsha, China) were used for total RNA extraction and reverse transcription following the manufacturer’s instructions, respectively.

RNA concentrations were measured by a CFX96 system (Bio–Rad, Hercules, CA, USA) and the quality were checked by 1% (w/v) agarose gel electrophoresis. Detailed procedures were as follows: preheating at 95 °C for 30 s, 40 cycles of 95 °C for 5 s, 60 °C for 30 s, and an increase of 0.5 °C every 5 s from 60 to 95 °C. The lactate dehydrogenase gene (*ldh*) was chosen as the internal reference gene. The mRNA levels of each target gene transcribed by the wild-type strain in MRS medium were set to 1 (with an OD_600_ value of 0.3). The relative expression levels of different target genes were calculated according to a previously reported method (Livak and Schmittgen 2001).

### Measurement of intracellular GTP

The cells harvested by centrifugation at 4 °C (8000 *g*, 10 min), then washed three times with ice-cold saline (0.85% NaCl, w/v), the detection of the intracellular GTP content was performed using a GTP Elisa Kit (AMOY LUNCHANGSHUO BIOTECH, CO., LTD, Xiamen, China) according to the manufacturer’s instructions.

### Statistical analysis

All statistical analyses were performed using IBM-SPSS Statistics 20.0 software (IBM Corp., Armonk, NY, USA). GraphPad Prism 8.0 (GraphPad Software, San Diego, CA, USA) and Adobe Illustrator CS6 (Adobe Systems Inc., San Jose, CA, USA) were used to draw graphs and typesets, respectively. Pearson correlation analysis was performed using R package ggcorrplot.

## Supplementary Information

**Additional file 1: Table S1.** Strains and plasmids used in this study. **Table S2.** Primers employed by this study. **Fig. S1.** Effects of differnent organic acid stress onthe survival rates of the wild-type XJ25 and the mutant XJ25Δ*mtp *cells in CDM. **Fig. S2.** The mRNA levels of *met1*, *met2*, *met3* in the acid-stressed cells of *L. plantarum* XJ25.

## Data Availability

All data generated or analyzed during this study are included in this published article.

## Abbreviations

MLF: Malolactic fermentation
LAB: Lactic acid bacteria
CDM: Chemically defined medium
SFAs: Saturated fatty acids
UFAs: Unsaturated fatty acids
