# Supplementary material for: Methionine addition improves the acid tolerance of Lactiplantibacillus plantarum by altering cellular metabolic flux, energy distribution, lipids composition
Source: Stress Biol. 2022 Nov 14;2(1):48. doi: 10.1007/s44154-022-00072-z (PMC10441991; doi:10.1007/s44154-022-00072-z)
Supplement: Supplementary file 1 — Additional file 1: Table S1. Strains and plasmids used in this study. Table S2. Primers employed by this study. Fig. S1. Effects of differnent organic acid stress onthe survival rates of the wild-type XJ25 and the mutant XJ25Δmtp cells in CDM. Fig. S2. The mRNA levels of met1, met2, met3 in the acid-stressed cells of L. plantarum XJ25. [file 44154_2022_72_MOESM1_ESM.docx]

**SUPPORTING INFORMATION**

Methionine addition improves the acid tolerance of *Lactiplantibacillus plantarum* by altering cellular metabolic flux, energy distribution, lipids composition

Qiang Meng, ^1^**^‖^** Yueyao Li,^1^**^‖^** Yuxin Yuan,^1^ Shaowen Wu,^1^ Kan Shi,1,2,3,4,5* and Shuwen Liu 1,2,3,4,5*

**^‖^**These authors contributed equally to this work.

*Corresponding author

^1^ College of Enology, Northwest A&F University, Yangling 712100, Shaanxi, China

^2^ Shaanxi Engineering Research Center for Viti-Viniculture, Yangling 712100, Shaanxi, China

^3^ Viti-viniculture Engineering Technology Center of State Forestry and Grassland Administration, Yangling 712100, Shaanxi, China

^4^ Heyang Experimental and Demonstrational Stations for Grape, Northwest A&F University, Heyang 715300, Shaanxi, China

^5^ Ningxia Helan Mountain's East Foothill Wine Experiment and Demonstration Station, Northwest A&F University, Yongning 750104, Ningxia, China

Correspondence address：

Kan Shi, E-mail: [s.kan@nwafu.edu.cn](mailto:s.kan@nwafu.edu.cn)

Shuwen Liu, E-mail: liushuwen@nwsuaf.edu.cn

**Table and figure of contents**

| **Names** | **Page** |
| --- | --- |
| **Table S1.** Strains and plasmids used in this study | S3 |
| **Table S2.** Primers employed by this study | S5 |
| **Fig. S1.** Effects of differnent organic acid stress on the survival rates of the wild-type XJ25 and the mutant XJ25Δ*mtp* cells in CDM. | S8 |
| **Fig. S2.** The mRNA levels of *met1*, *met2*, *met3* in the acid-stressed cells of *L. plantarum* XJ25. | S9 |

**Table S1.** Strains and plasmids used in this study

| **Strains/ Plasmids** | **Description** | **Source/Reference** |
| --- | --- | --- |
| *E. coli* HST04 *dam^-^/dcm^-^* | cloning strain lacking two DNA methyltransferase genes *dam* and *dcm* | Takara |
| HST04/pLCNICK | *E. coli* DH5α derivative harboring pLCNICK | Laboratory stock |
| HST04/pLCNICK-Δ*mtp* | *E. coli* HST04 *dam^-^/dcm^-^* derivative harboring pLCNICK-Δ*mtp* | This study |
| HST04/pNZ8148 | *E. coli* HST04 *dam^-^/dcm^-^* derivative harboring pMG36ea | Meng et al., 2021 |
| HST04/pMG36ea | *E. coli* HST04 *dam^-^/dcm^-^* derivative harboring pNZ8148 | This study |
| HST04/pNZ-P_OL2_-*metK* | *E. coli* HST04 *dam^-^/dcm^-^* derivative harboring pNZ-P_OL2_-*metK* | This study |
| HST04/pNZ-P_OL2_-*metC* | *E. coli* HST04 *dam^-^/dcm^-^* derivative harboring pNZ-P_OL2_-*metC* | This study |
| HST04/pNZ-P_OL2_-*metB* | *E. coli* HST04 *dam^-^/dcm^-^* derivative harboring pNZ-P_OL2_-*metB* | This study |
| HST04/pMG-P_OL2_-*metC* | *E. coli* HST04 *dam^-^/dcm^-^*derivative harboring pMG-P_OL2_-*metC* | This study |
| HST04/pMG-P_OL2_-*metB* | *E. coli* HST04 *dam^-^/dcm^-^* derivative harboring pMG-P_OL2_-*metB* | This study |
| *L. plantarum* XJ25 | wild type strain, isolated from Chinese red wine | Laboratory stock |
| XJ25/pNZ8148 | *L. plantarum* XJ25 derivative harboring pNZ8148 | Meng et al., 2021 |
| XJ25/pMG36ea | *L. plantarum* XJ25 derivative harboring pMG36ea | This study |
| XJ25/pNZ-P_OL2_-*metK* | *L. plantarum* XJ25 derivative harboring pNZ-P_OL2_-*metK* | This study |
| XJ25/pNZ-P_OL2_-*metC* | *L. plantarum* XJ25 derivative harboring pNZ-P_OL2_-*metC* | This study |

| **Table S1.**(continued) |  |  |
| --- | --- | --- |
| **Strains/ Plasmids** | **Description** | **Source/Reference** |
| XJ25/pNZ-P_OL2_-*metB* | *L. plantarum* XJ25 derivative harboring pNZ-P_OL2_-*metB* | This study |
| XJ25/pMG-P_OL2_-*metC* | *L. plantarum* XJ25 derivative harboring pMG-P_OL2_-*metC* | This study |
| XJ25/pMG-P_OL2_-*metB* | *L. plantarum* XJ25 derivative harboring pMG-P_OL2_-*metB* | This study |
| **Plasmids** |  |  |
| pLCNICK | *repD*, *repE*, *repA101*, P_23_-Cas9^D10A^ expression cassette, P*_ldh_* | Song et al., 2017 |
| pLCNICK-Δ*mtp* | pLCNICK derivative, the Δ*mtp*-sgRNA fragment was inserted between the *Apa* I and *Xba* I sites | This study |
| pMG36e | pWV01 *ori*, constitutive promoter P_32_, *L. lactis*-*E. coli* shuttle vector | Laboratory stock |
| pMG36ea | pMG36e derivative, an ampicillin resistance gene was inserted downstream the erythromycin resistance gene | This study |
| pNZ8148 | *repA*, *repC*, P*_nisA_*-*Nco*I-multiple cloning sites-terminator (*Nco* I site employed for translational fusions) | Reference |
| pNZ-P_OL2_-*metK* | pNZ8148 derivative, the P*_nisA_* promoter was replaced with the P_OL2_-*metK* expression cassette | This study |
| pNZ-P_OL2_-*metC* | pNZ8148 derivative, the P*_nisA_* promoter was replaced with the P_OL2_-*metC* expression cassette | This study |
| pNZ-P_OL2_-*metB* | pNZ8148 derivative, theP*_nisA_* promoter was replaced with the P_OL2_-*metB* expression cassette | This study |
| pMG-P_OL2_-*metC* | pMG36ea derivative,the P_32_ promoter was replaced with the P_OL2_-*metC* expression cassette | This study |
| pMG-P_OL2_-*metB* | pMG36ea derivative,the P_32_ promoter was replaced with the P_OL2_-*metB* expression cassette | This study |

**Table S2.** Primers employed by this study

| **Primers** | **Sequence (5'→3')** |
| --- | --- |
| mtp-uF | GGTGCTTTTTTTGAGCGATGATTCCTTGGAGACGCG |
| mtp-uR | CATCTTAGTAATTCTCCTTCTCTATCAATC |
| mtp-dF | AGGAGAATTACTAAGATGTAATCGGCTTAGTATCAACAAATTAATTGTC |
| mtp-dR | TCTTTTTCTAAACTAGGGCCCCAGTACTTGCCGCTACGC |
| sg-F | CTCAAAAAAAGCACCGACTC |
| sg-mtp-R | AGGATGATATCACCTCTAGAAAATGCCACTATCAGTTGGGGTTTTAGAGCTAGAAATA |
| inser-uF | TCTTTTTCTAAACTAGGGCCCCAGTAC |
| inser-uR | TCGGCTTAGTATCAACAAATTAATTGTC |
| inser-dF | CTTAGTAATTCTCCTTCTCTATCAATCA |
| inser-dR | AAGGATGATATCACCTCTAGAAAATGCC |
| pNZ-zt-F | AAGCTTTCTTTGAACCAA |
| pNZ-zt-R | ATTCATATTTTTCATCTCGAATATCCTCC |
| pNZ-metK-F | ATGAAAAATATGAATGTGAGTGAAAGACACTTATTTACATC |
| pNZ-metK-R | GTTCAAAGAAAGCTTTTATTTAAATGCTGCTTTTAAAGCATCC |
| pNZ-metC-F | ATGAAAAATATGAATATGACAACAACGAATCCTG |
| pNZ-metC-R | GTTCAAAGAAAGCTTTTAAACCTGTGCCAATGCTTG |
| pNZ-metB-F | ATGAAAAATATGAATATGACAAAACAAGCTGAG |
| pNZ-metB-R | GTTCAAAGAAAGCTTTTATTTTGTTGCTCGGATCAGCG |

| **Table S2.**(continued) |  |
| --- | --- |
| **Primers** | **Sequence (5'→3')** |
| pMG-zt-F | AAGCTTTGCAAAGTCTGAAAACGAAGG |
| pMG-zt-R | ATCCCGAGGACCGAATTCGATCG |
| pMG-metC-F | TTCGGTCCTCGGGATCGAAAAGCCCTGACAACC |
| pMG-metC-R | GACTTTGCAAAGCTTTTAAACCTGTGCCAATGCTTG |
| pMG-metB-F | TTCGGTCCTCGGGATATGACAAAACAAGCTGAGAAATTACAC |
| pMG-metB-R | GACTTTGCAAAGCTTTTATTTTGTTGCTCGGATCAGCG |
| q-mtp-F | GGTTACGGTTGCCATTTACGTCGCGACG |
| q-mtp-R | CCGTGTAAATCACCAACAATCGTTGGCAAGGC |
| q-metK-F | GTCGTGCGTGACACGATCAAGTCGATTGGC |
| q-metK-R | CGCCAGAACGCGTCTCCAAGGAATCATCG |
| q-1210-F | CTGTTGATGGGCCGAGCATCATTGAGGTCG |
| q-1210-R | GCTCGCAATGCGTCCTGATGCGGAACG |
| q-7955-F | CGGCTCACCAGATTGGCGATACTGATCC |
| q-7955-R | TGGACTGCCCAGGATGTCCTGGACATGG |
| q-9410-F | CTTCCGCAGCCCGTTGTAATCGTTGACTC |
| q-9410-R | GGCCGAAATCCAAGTCCGGACACTTGCC |

| **Table S2.**(continued) |  |
| --- | --- |
| **Primers** | **Sequence (5'→3')** |
| q-cblB-F | GCCAGAAGCAAATGCGAAGCCAGCGCTG |
| q-cblB-R | CCCATCTACATGGCCTCGACCTTCCGCC |
| q-metC-F | GCGAGCGTTACCCCAGCCGTCCCGTGC |
| q-metC-R | GGATGCCAAGCAAGCTGCGGGTCGC |
| q-metB-F | CGAGCTGGATTGCCGCCATGCCAGAAG |
| q-metB-R | ATCTACAGGATTTGACTACCCGCGCGAG |
| q-ldh-F | TTGGTGATGGCTCGGTGGGTTCATCA |
| q-ldh-R | TCGCATTCGTAAAGGCGGCAACATCTTCC |


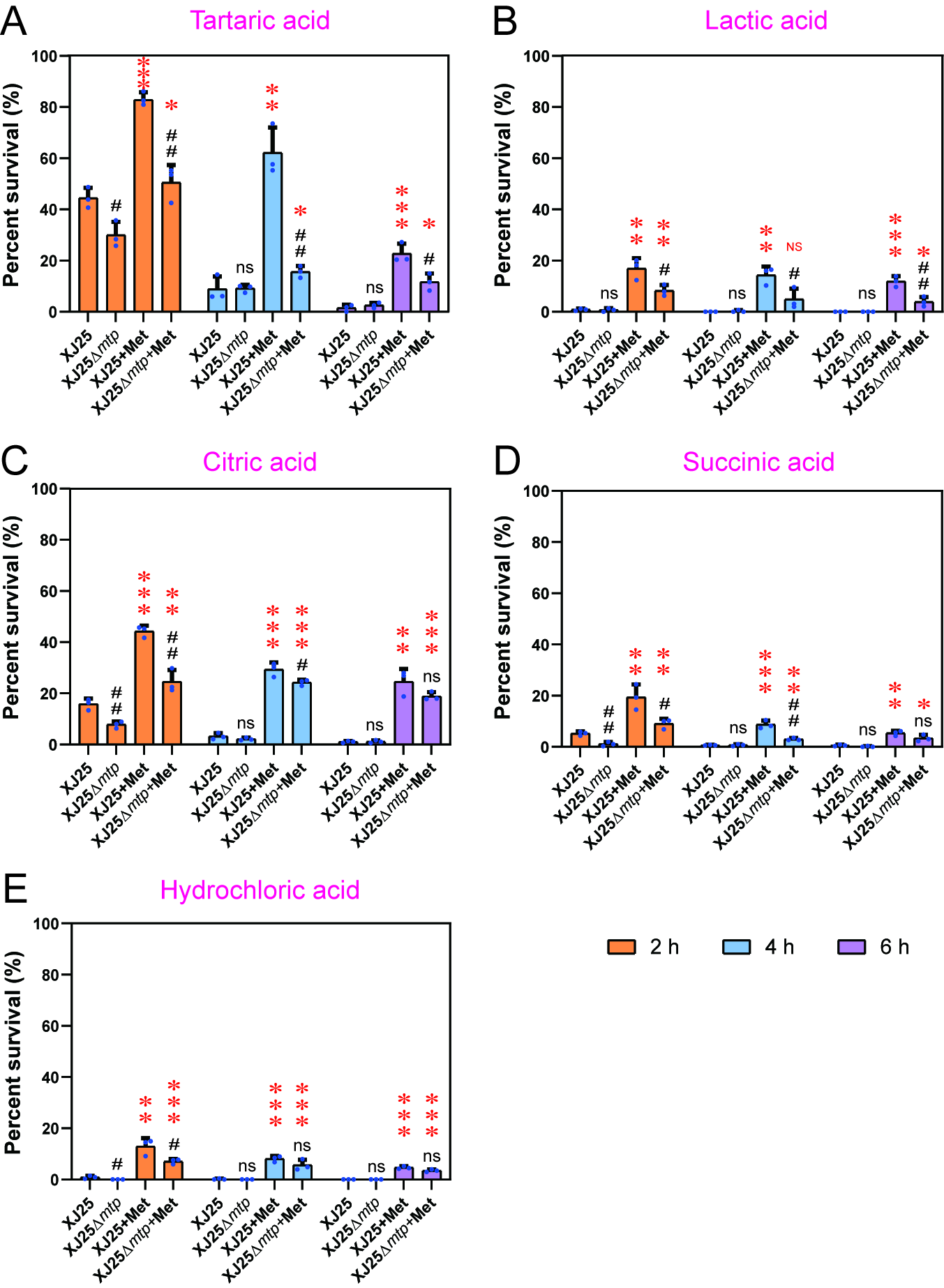


**Fig. S1.** Effects of differnent acid stress on the survival rates of the wild-type XJ25 and the mutant XJ25Δ*mtp* cells in CDM. Tartaric acid (A); lactic acid (B); citric acid (C); succinic acid (D); hydrochloric acid (E). + Met, 2 mM methionine. ^#^ *p*< 0.05, ^##^ *p*< 0.01, ns, not significant (*p*> 0.05), comparison between the wild-type XJ25 and the mutant XJ25Δ*mtp* (XJ25 vs XJ25Δ*mtp*, XJ25 + Met vs XJ25Δ*mtp* + Met, Student’s *t*-test); * *p*< 0.05, ** *p*< 0.01, *** *p*< 0.001, NS, not significant (*p*> 0.05), comparison between the stressed cells supplied with and without methionine (XJ25 vs XJ25 + Met, XJ25Δ*mtp* vs XJ25Δ*mtp* + Met, Student’s *t*-test).


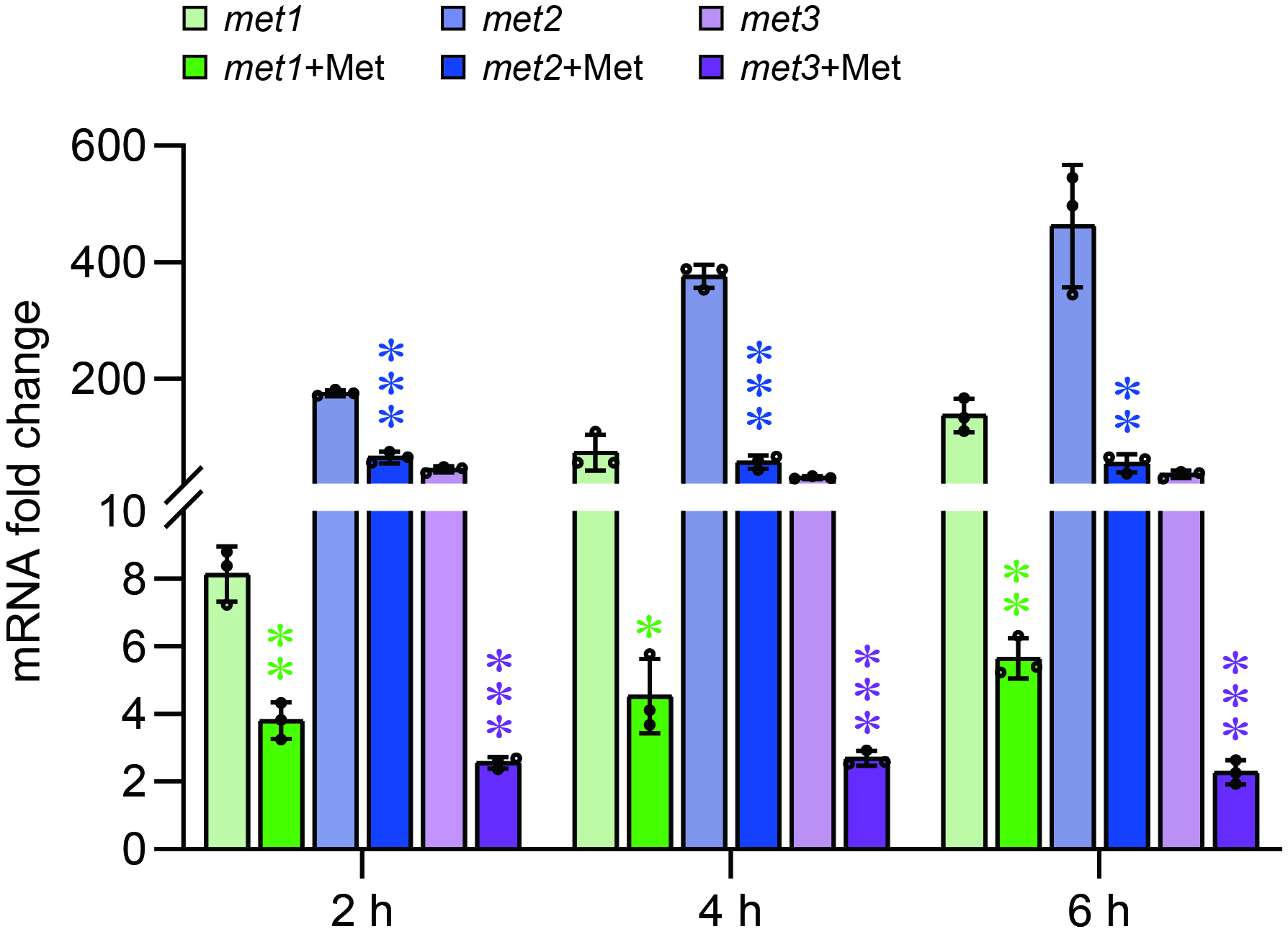


**Fig. S2.** The mRNA levels of *met1*, *met2*, *met3* in the acid-stressed cells of *L. plantarum* XJ25. + Met, 2 mM methionine. Three putative *met* operons were found in the genome of XJ25 using HMMER package v3.1b1, namely *met1*, *met2* and *met3*. The *JKL54_RS01435* mRNA level (relative to the internal reference gene *ldh*) in the wild-type XJ25 (with an OD600 value of ~0.3) in MRS medium (pH 6.0) was set to 1. * *p*< 0.05, ** *p*< 0.01, *** *p*< 0.001, comparison between the treatments with and without methionine supplementation (*met1* vs *met1* + Met, *met2* vs *met2* + Met, *met3* vs *met3* + Met, Student’s *t*-test).
